# Posterior instrumented fusion on lumbar stenosis syndrome can bring benefit to proximal degenerative kyphosis: A CONSORT-compliant article

**DOI:** 10.1097/MD.0000000000027711

**Published:** 2021-11-12

**Authors:** Shuai Xu, Chen Guo, Yan Liang, Zhenqi Zhu, Hongguang Zhang, Haiying Liu

**Affiliations:** a Department of Spinal Surgery, Peking University People's Hospital, Peking University, Beijing, P.R. China; b Department of Neurosurgery, Jining Medical College Affiliated Gaotang People's Hospital, Liaocheng, Shandong, P.R. China.

**Keywords:** degenerative thoracolumbar kyphosis, lower instrumented vertebrae, lumbar spinal stenosis syndrome, osteoporosis, pelvic incidence, pelvic tilt, sacral slope, short-level fixation, spino-pelvic alignment

## Abstract

The effect on degenerative thoracolumbar kyphosis (DTLK) after short-segment instrument for lumbar spinal stenosis syndrome (LSS) remains controversial. Based on the biomechanics and compensatory of the global spino-pelvic alignment, it was assumed that the interference on the lumbar spine, instead of the thoracolumbar segment, could still make a difference on the proximal spine.

To explore whether DTLK could improve with only surgery for LSS and identify influencing factors on postoperative TLK.

The study was performed from January 2016 to December 2018. Sixty-nine participants (25 male) diagnosed LSS with DTLK were enrolled and surgery was only for LSS. Radiological parameters included TLK, lumbar lordosis, pelvic incidence, pelvic tilt, sacral slope, and osteoporosis. Clinical outcomes were visual analogue scale and Oswestry disability index. According to lower instrumented vertebrae (LIV) on L5 or S1, inter-group comparisons were performed between LIV on L5 (L5 group) and S1 (S1 group).

Demographics were well-matched between L5 and S1 group with a mean follow-up of 24.3 ± 12.1 (m). TLK improved with a mean of 16.2 ± 7.6 (°) (*P* < .001). There was no significance on radiological and clinical parameters between L5 and S1 groups except for a larger pelvic tilt in S1 group (*P* = .046). Visual analogue scale (*P* = .787) and Oswestry disability index (*P* = .530) were both indifferent between normal TLK and DTLK at last (*P* > .05). Postoperative TLK was affected by osteoporosis and sacral slope, the latter was dominated by pelvic incidence and pelvic rotation. Osteoporosis was the risk factor for TLK correction (*P* = .001, odd risk = 9.58).

DTLK decreased if instrument only performed for LSS, where TLK and clinical outcomes are comparably affected whether L5 or S1 is selected as LIV. This study supplements the compensatory mechanism of spino-pelvic alignment, especially for cases with severe osteoporosis.

## 1 Introduction

Degenerative thoracolumbar kyphosis (DTLK), as a kind of adult spinal deformity, is a common degenerative spinal disease in the elderly.^[1]^ Lumbar spinal stenosis syndrome (LSS) combined with DTLK, with gradually increasing exposure rate nowadays, can result in low back pain and lower extremities dysfunction,^[2]^ which is sometimes cured by surgery, and the majority of the series mind the symptom of LSS instead of DTLK.^[3]^ There is no doubt that it is essential to perform decompression and fixation for responsible levels with severe LSS, disc protrusion, or instability, while whether implanting instruments and perform deformity correction referring to the region of TLK is always filled with controversy.^[4,5]^

It is reported much success has been achieved on TLK deformity through 1 to 3 grade osteotomy and long-segment instrument spanning across this region, while adverse outcomes have emerged such as extensive trauma and operating duration, huge expenses, and even instrument-related infection.^[6,7]^ Recently, more and more experienced surgeons proposed the definition of short-segment fixation and the theory of precision therapy,^[8,9]^ where the chief complaints were adequate to solve rather than correcting all malformation into a reasonable range. Shin et al^[10]^ suggested simple decompression surgery, not the disordered levels, was able to restore sagittal alignment in 70% of patients caused by LSS, but the point was not further identified on LSS combined with DTLK yet.

There is biomechanical interaction among sagittal parameters of spino-pelvic alignment, where thoracolumbar segments, as a bridge element, is probably affected by other regions.^[1,11]^ In addition, once S1 was selected as lower instrumented vertebrae (LIV), the sacral slope (SS) and pelvic tilt (PT) was unchangeable since the pelvic incidence (PI) was almost fixed in the adult with PI = PT + SS. Theoretically, the parameters may be statistically different when L5 was determined as LIV with more range of pelvic rotation.^[12,13]^ While it is unclear whether TLK can be influenced by L5 or S1 as LIV in short-segment fusion through biomechanical chain.

Based on the compensatory mechanism of the global spino-pelvic alignment, it was assumed that the interference on the lumbar spine, instead of the thoracolumbar segment, could still make a difference on the proximal spine, which may supplement the theory of dynamic balance of sagittal alignment and support the evidence for LIV selection. Therefore, the study included patients diagnosed as LSS combined with DTLK, who were performed short-segment fixation simply for LSS, where upper instrumented vertebrae was lower than L2. A short-term follow-up was conducted to identify whether DTLK could improve after the instrument within lumbar spine, whether the selection of LIV on L5 or S1 can make a difference on proximal thoracolumbar spine, and the risk factors on the correction of proximal segments after surgery only on LSS.

## 2 Materials and methods

### 2.1 Patients enrollment

The single-center retrospective protocol was performed from January 2016 to December 2018. The participants diagnosed LSS combined with DTLK was enrolled. The study was approved by local institutional review board and all patients have signed informed consent.

The inclusion criteria included the patients were older than 55 years; patients were diagnosed as LSS with surgery indication; patients suffered from sagittal TLK resulted definitely from degeneration (TLK ≥15°); local fixation for LSS: upper instrumented vertebrae was not above L2 and LIV was L5 or S1; intact stand lumbar and whole spine X-ray and lumbosacral computed tomography (CT) and magnetic resonance imaging (MRI) could be obtained; it was primary surgery; and without coronal deformity. The exclusion criteria were LSS not reached surgical-indication yet; TLK due to non-degenerated deformity such as ankylosing spondylitis, compressed fracture, or Scheuermann disease; with coronal deformity; inner-fixation referred to or stretched across thoracolumbar levels; incomplete or unclear radiological data for measurement; secondary or revised surgery; and loss of follow up.

For DTLK cases, posterior lumbar interbody fusion (PLIF) and posterior-lateral fusion was applied mainly for lumbar segments with spinal stenosis, disc hernia, and instability, which was instrumented in-situ or with grade 1 to 3 osteotomy. All patients were operated on by 1 senior surgeon.

### 2.2 Radiological parameters and clinical outcomes

**Figure 1:**
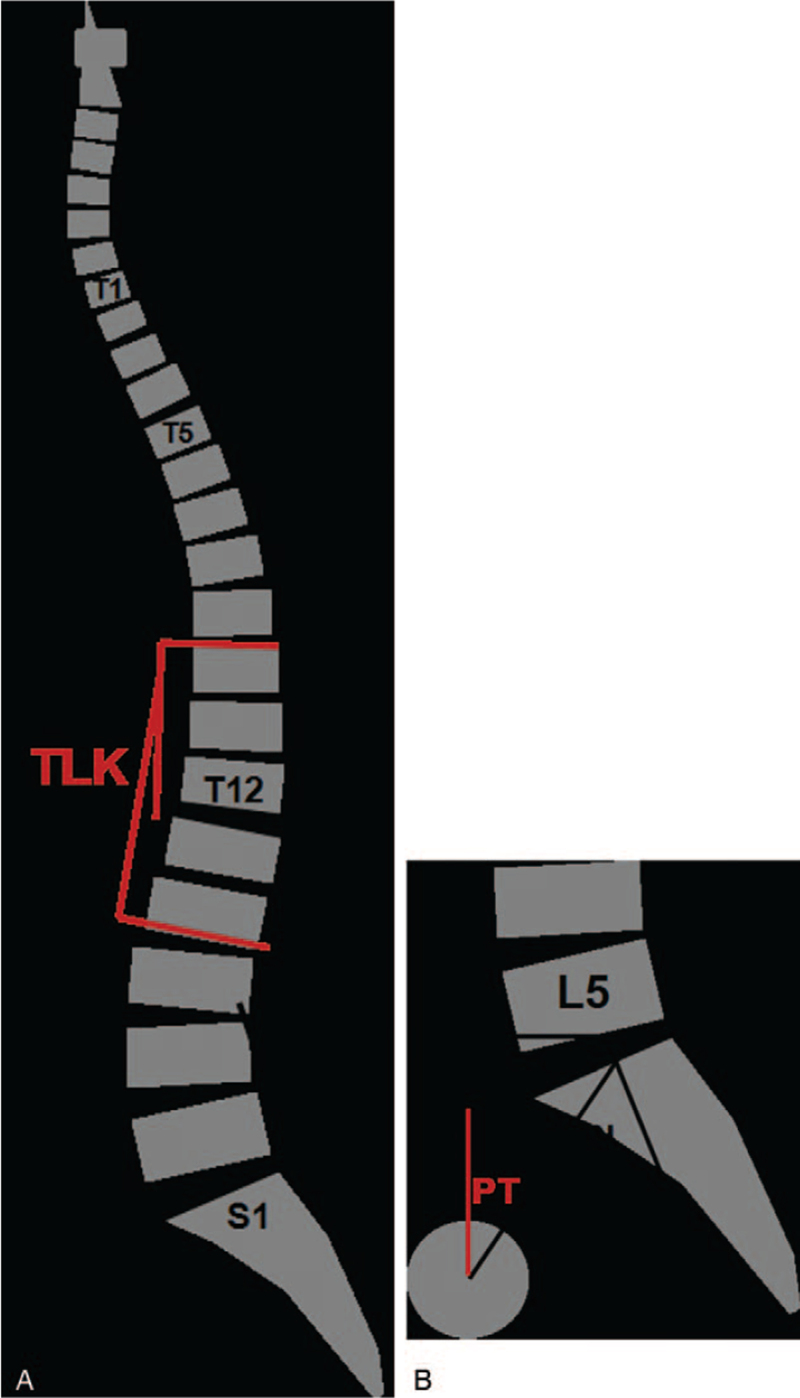
Diagram of spino-pelvic radiological parameters. (A) TK was the angle between upper endplate of T5 and lower endplate of T12. TLK was the angle between upper endplate of T10 and lower endplate of L2. LL was between upper endplate of L1 and upper endplate of S1. (B) PT was the angle between plumb line and the center of the femoral head to midpoint of upper endplate of S1. PI was the vertical line passing through the midpoint of upper endplate on S1, then second line connecting midpoint of the upper endplate on S1 and the femoral head and the angle between the second line and vertical line. SS was the angle between upper endplate of S1 and horizontal line. LL = lumbar lordosis, PI = pelvic incidence, PT = pelvic tilt, SS = sacral slope, TK = thoracic kyphosis, TLK = thoracolumbar kyphosis.

Radiological parameters included kyphosis apex, thoracic kyphosis (TK), TLK, lumbar lordosis (LL), PI, PT, and SS. TK was the angle between upper endplate of T5 and lower endplate of T12; TLK was the angle between upper endplate of T10 and lower endplate of L2; LL was between upper endplate of L1 and upper endplate of S1. The definition for PI, PT, and SS was shown in Figure 1. Osteoporosis was determined by CT, MRI, and surgical records, where severe osteoporosis was manifested by decreased bone density, thinned trabecular bone, biconcave or wedge-shaped changes in vertebrae, which was together evaluated during operation.^[14]^

Clinical outcomes were evaluated by visual analogue scale (VAS) and Oswestry disability index (ODI). VAS ranged from 0 to 10 and a higher score implied more severe pain. ODI reflected disability on lumbar spine function and quality of life (0–50 score) and higher index represented more disability. All outcomes were measured before operation (baseline) and at follow-up endpoint.

Furthermore, according to LIV on L5 or S1, inter-group comparisons were performed between LIV on L5 (L5 group) and S1 (S1 group).

### 2.3 Statistical analysis

The measurement data were depicted as mean ± standard deviation. The dichotomous between groups were analyzed by χ^2^ test. Independent sample *t* test was used for inter-group measurement data and paired *t* test was applied between baseline and endpoint, while Wilcoxon test was used for ordinal data. Pearson or Spearman correlation analysis was used among parameters and between TLK and demographics. Multiple linear regression and logistic regression were for determining the influencing factors of TLK. SPSS 22.0 (IBMC, Armonk, NY) the software for statistical analysis and *P* < .05 was significant difference.

## 3 Results

A total of 69 DTLK patients (25 male) was included with a mean follow-up of 24.3 ± 12.1 (m), the age and body mass index (BMI) were respectively 68.9 ± 9.2 (55–84) (yr) and 26.1 ± 3.5 (kg/m^2^). The most operated segments was L2–L5 (29.0%), followed by L3–L5 (24.6%) and L3–S1 (14.5%). There were no significances in gender, age, and BMI between L5 group and S1 group, so was osteoporosis (*P* > .05). The number of operated segments was larger in S1 group (*P* < .05) (Table 1).

**Table 1:** Basic information between L5 group and S1 group of DTLK.

Statistics	L5 group	S1 group	*P*
Gender (M:F)	17:27	8:17	.581
Age (yrs)	69.1 ± 8.7	67.5 ± 10.2	.494
BMI (kg/m^2^)	25.9 ± 3.2	26.6 ± 4.0	.422
Osteoporosis	21	12	.983
No. of screws	6.5 ± 1.5	7.7 ± 1.7	.006
No. of segments	2.3 ± 0.8	2.8 ± 0.9	.006
Follow up (m)	24.2 ± 12.1	24.6 ± 12.5	.899

BMI = body mass index, DTLK = degenerative thoracolumbar kyphosis, F = female, M = male.

**Figure 2:**
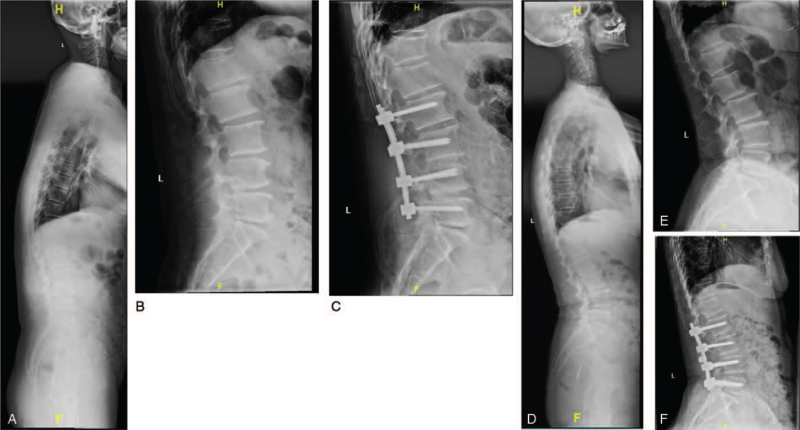
Cases on PLIF only for responsible levels with LSS, instability and disc hernia in DTLK patients. (A–C) 67 year-old male, he was performed L2-L5 PLIF with a follow-up of 26 months. At baseline, TK = 12.8, TLK = 24.9, LL = 17.8, PI = 29.3, PT = 4.2, SS = 25.1, VAS = 9, and ODI = 44. At the endpoint, TK = 10.5, TLK = 16.7, LL = 18.9, PI = 29.0, PT = 6.6, SS = 22.4, VAS = 1, and ODI = 6. (D–F) 69 year-old female, she was performed L3-S1 PLIF with a follow-up of 23 months. At baseline, TK = 33.9, TLK = 20.4, LL = 52.0, PI = 44.4, PT = 11.3, SS = 33.1, VAS = 6, and ODI = 45. At the endpoint, TK = 35.6, TLK = 9.8, LL = 47.9, PI = 43.6, PT = 15.4, SS = 28.2, VAS = 1, and ODI = 6. Although there was posterior olisthesis of L2, L2-L3 kept in stability at the endpoint. DTLK = degenerative thoracolumbar kyphosis, LL = lumbar lordosis, LSS = lumbar spinal stenosis syndrome, ODI = Oswestry disability index, PI = pelvic incidence, PLIF = posterior lumbar interbody fusion, PT = pelvic tilt, SS = sacral slope, TK = thoracic kyphosis, TLK = thoracolumbar kyphosis, VAS = visual analogue scale.

At the endpoint, 63 cases (91.3%) got a decrease on TLK by a mean of 8.9 ± 11.5 (°) and 27 patients acquired normal TLK. Thus, TLK improved with a mean of 16.2 ± 7.6 (°) compared to baseline (*P* < .001), so did TK (*P* = .022). While there were no statistical differences on LL, PI, PT, and SS between the 2 time points (*P* > .05). In addition, VAS and ODI both decreased (1.9 ± 1.6 and 7.9 ± 6.3, respectively) at last (both *P* < .001) (Table 2). At baseline, there were no significances on radiological parameters between L5 and S1 group, so were VAS and ODI (*P* < .05). These parameters were also comparable at the endpoint between groups except a larger PT (22.5 ± 8.6) in S1 group (*P* = .046). In L5 group, TK (20.7 ± 12.1) and TLK (16.5 ± 7.3) decreased at final compared to baseline (*P* = .041 and *P* = .001, respectively) while the others kept stable, the same situation in S1 group (*P* = .047 and *P* = .012, respectively) (Table 3) (Fig. 2).

**Table 2:** Sagittal parameters and clinical outcomes between baseline and endpoint.

	Pre-operation	Endpoint	*P*
TK (°)	32.3 ± 15.8	22.4 ± 12.5	.022
TLK (°)	24.9 ± 8.3	16.2 ± 7.6	<.001
LL (°)	38.3 ± 18.1	40.1 ± 14.1	.217
PI (°)	46.2 ± 11.7	46.7 ± 10.4	.630
PT (°)	19.7 ± 8.1	19.4 ± 9.8	.736
SS (°)	26.6 ± 10.9	27.3 ± 9.5	.527
VAS	6.6 ± 1.4	1.9 ± 1.6	<.001
ODI	35.8 ± 6.9	7.9 ± 6.3	<.001

LL = lumbar lordosis, ODI = Oswestry disability index, PI = pelvic incidence, PT = pelvic tilt, SS = sacral slope, TK = thoracic kyphosis, TLK = thoracolumbar kyphosis, VAS = visual analogue scale.

**Table 3:** Sagittal parameters and clinical outcomes between L5 and S1 group.

	Pre-operation			Endpoint		
	L5 group	S1 group	*P*	L5 group	S1 group	*P*
TK (°)	31.0 ± 15.8	34.3 ± 15.9	.427	20.7 ± 12.1^∗^	24.4 ± 13.8^∗^	.577
TLK (°)	25.9 ± 9.1	23.2 ± 6.2	.189	16.5 ± 7.3^†^	15.8 ± 8.3^∗^	.760
LL (°)	36.8 ± 16.0	40.7 ± 20.5	.386	39.6 ± 14.0	41.0 ± 14.5	.691
PI (°)	45.0 ± 11.3	47.9 ± 12.0	.305	46.1 ± 10.7	47.9 ± 9.9	.506
PT (°)	19.2 ± 7.1	20.5 ± 9.4	.453	17.7 ± 10.0	22.5 ± 8.6	.046
SS (°)	26.1 ± 10.0	27.4 ± 12.0	.638	28.3 ± 9.4	25.3 ± 9.6	.225
VAS	6.6 ± 1.4	6.6 ± 1.3	.937	1.9 ± 1.4	1.8 ± 1.9	.851
ODI	35.2 ± 7.7	37.0 ± 5.1	.363	7.7 ± 5.1	8.3 ± 8.1	.706

LL = lumbar lordosis, ODI = Oswestry disability index, PI = pelvic incidence, PT = pelvic tilt, SS = sacral slope, TK = thoracic kyphosis, TLK = thoracolumbar kyphosis, VAS = visual analogue scale.

∗ There was statistical significance between baseline and endpoint in the same group (*P* < .05).

† There was statistical significance between baseline and endpoint in the same group (*P* < .01).

Many radiological parameters were highly correlated to each other at the endpoint while TLK was just negatively to PI (r = –0.340, *P* = .010) and SS (r = –0.415, *P* = .001) (Table 4). There were no relationships between TLK and VAS (*P* = .678) or ODI (*P* = .786) at last. What's more, VAS (*P* = .787) and ODI (*P* = .530) were both of no significances between ones with normal TLK and DTLK series at last. TLK was not related to gender (*P* = .973), age (*P* = .749), and BMI (*P* = .636) at last, but positively correlated to osteoporosis (r = 0.343, *P* = .009).

**Table 4:** Correlation analysis between TLK and radiological or clinical parameters at endpoint.

	TK		TLK		LL		PI		PT		SS		VAS	
	r	*P*	r	*P*	r	*P*	r	*P*	r	*P*	r	*P*	r	*P*
TLK	−0.206	.445												
LL	0.576	.019	−0.213	.112										
PI	0.136	.615	−0.340	.010	0.441	<.001								
PT	−0.338	.200	0.031	.817	−0.248	.024	0.555	<.001						
SS	0.364	.166	−0.415	.001	0.732	<.001	0.523	<.001	−0.418	<.001				
VAS	0.079	.828	0.062	.678	−0.073	.593	0.030	.827	0.081	.551	−0.064	.640		
ODI	0.175	.630	−0.040	.786	−0.044	.746	0.135	.320	0.216	.110	−0.096	.481	0.848	<.001

LL = lumbar lordosis, ODI = Oswestry disability index, PI = pelvic incidence, PT = pelvic tilt, SS = sacral slope, TK = thoracic kyphosis, TLK = thoracolumbar kyphosis, VAS = visual analogue scale.

TLK at the endpoint was regarded as a dependent variable, where parameters with *P* < .2^[15]^ in correlation analysis were included as independent variables. Table 5 showed lower SS and severe osteoporosis were risk factors for postoperative TLK (Beta = –0.427, *P* = .025 and Beta = 0.374, *P* = .039) (Table 5). SS was determined by PI and PT, where SS = PI-PT. When postoperative TLK was seen as dichotomous by corrected TLK and DTLK, logistic regression was performed. When LL, PI, SS, and severe osteoporosis were pooled, it showed severe osteoporosis was the independent risk factor for TLK correction (*P* = .001, χ^2^ = 14.97, odd risk (OR) = 9.58, 95% confidence interval [2.49,36.93]).

**Table 5:** Multiple linear regression analysis of TLK at endpoint.

	Unstandardized		Standardized		
Coefficient	B	SE	Beta	T	*P*
(Constant)	27.336	4.561		5.993	.000
LL	0.111	0.095	0.202	1.158	.252
PI	−0.122	0.106	−0.170	−1.147	.256
SS	−0.343	0.149	−0.427	−2.306	.025
Osteoporosis	2.612	1.871	0.374	2.396	.039

LL = lumbar lordosis, PI = pelvic incidence, SE = standard error, SS = sacral slope, TLK = thoracolumbar kyphosis.

## 4 Discussion

DTLK, as a kind of adult spinal deformity (ASD), has been one of the hot topics in the field of spine surgery with the incidence of 5% to 34%.^[7]^ The majority of the population have no obvious clinical manifestations with occasionally mild low back pain and paraspinal muscle tension.^[1]^ Only a small group visits the doctors with severe restriction on quality of life and sagittal malalignment, let alone the proportion of surgical-demanding. Although increasing requirement on body shape, it may be undeserved to perform orthopedic with huge cost, enlarged invasion while lower effectiveness, especially for the middle-aged and elderly.^[16]^ Due to the shorter rest-survival expectation, self-regulation or conservative treatment is usually adopted for the most. However, as the most common degenerative spinal disease in this group, LSS can induce lower extremities pain and intermittent claudication with poor quality of life and there is no doubt that surgical treatment on responsible segment is chosen for most cases with severe LSS.^[10]^ The population diagnosed LSS combined with DTLK is non-ignorable based on magnanimous LSS cases, where they often wonder whether DTLK needs to be corrected since the chief complaints are mainly caused by LSS. It is also too confusing for many spinal surgeons to give the identified answer.

Surgical correction with osteotomy and long-segment fusion on TLK has proven to be effective, leading to superior body appearance, clinical and radiographic outcomes, especially proper alignment is restored.^[7,17]^ However, surgical treatment spanning across malformed TLK remains challenging as demonstrated by revisions (9.0%–17.6%) and adverse events such as extensive tissue stripping, burden expenses, and rod fracture.^[18,19]^ With the concept of minimum of invasion and maximum of efficacy, the superiority of precision therapy and short-level instrument are promoting, particularly with more profound knowledge of ASD, qualified implants with better biocompatibility and Young modulus, improved technology and perioperative management.^[20]^ Once lower complications ratio and effective quality of life is still achieved with decompression and fixation only on responsible level, it will be significant in saving marvelous wealth for society, government, and themselves. This study was retrospectively performed on cases with LSS and DTLK, where the series underwent PLIF or PIF only for LSS, not referring to TLK. In total, it showed effective outcomes although there were 4 patients (5.8%) in instability on upper adjacent segment and 2 cases (2.9%) in proximal junctional kyphosis while with no symptom at endpoint (data not shown), which proved the concept of “precision fixation” for DTLK ones was appropriate.

Patients with LSS appear with lumbar extension and improve with trunk flexion, where they can take severely stooped posture aiming pain relief.^[18]^ Fujii et al^[21]^ observed decompression and proper fixation in LSS was followed by improvement in alignment parameters such as reduction of PT and TK and increase of LL, the same point of Shin et al.^[10]^ According to Redaelli et al's point,^[18]^ 2 different categories on TLK was divided: structural and non-structural TLK, the latter acted like inducing adaptation on alignment with reversibility, which was considered compensatory TLK due to focal pathologies. LSS can behave as a focal cause of DTLK, but the way to distinguish the 2 subsets categories is not clear.^[22]^ In our data, TLK ranged from 15.2° to 45.5° at baseline and most was regarded as non-structural TLK by radiological measurement and intra-operative judgement.

This study observed DTLK cases acquired improvement on TLK with only treatment for LSS with a mean TLK reduction by about 10° and normal TLK achieved near to 40%. On the one hand, adequate decompression of LSS removed pain derived from nerve root compression and ensuing reduced the trunk anteversion.^[23]^ On the other hand, the recovery and stability of LL optimized compensation especially on proximal TK and TLK with the motion preserving.^[10]^ In addition, the paraspinal muscles got stronger by cooperating with functional exercise after surgery, which probably reduced TLK.^[24]^

Sagittal alignment, biomechanism, and clinical outcomes have been discussed when L5 or S1 was respectively chosen as LIV. Yasuda et al^[25]^ found fusion to L5 was conducted for selected ASD patients with better ODI and less complex deformity contrasted with S1 as LIV. Choi and Jeon^[13]^ identified S1 double screws are a viable option for sacropelvic fixation in ASD patients when L5 pedicle screw fixation was not possible, and S1 group achieved better reconstruction on LL while restricted pelvis rotation. In this study, the only difference between L5 and S1 group was whether L5-S1 was etiological segment. The clinical outcomes were comparable in L5 and S1 at endpoint but the pelvic retroversion compared to L5 group, which was not consistent to Yasuda et al while agreed with Choi and Jeon. Although the restricted motion of L5-S1 in S1 group, the compensatory of proximal segment played an important role, which made comparable results on alignments between the 2 groups.^[26]^ In addition, both groups acquired decreased TLK and TK at last, mainly because the removal of compression and enlargement of lumbar canal, consequently the stooped posture for pain-relief was corrected,^[2,17]^ which was in line with Fujii et al.

There are interaction among alignment parameters but TLK seems like estranged from others. It is considered that the abnormality of the transition site is affected by many aspects. Thoracolumbar region locates in the transitional part of both anatomical and biomechanical structure with large shear force, where vertebrae or intervertebral discs involved in the region are prone to wedge-shaped. Then, fibrosis of the anterior ligament force it closer between adjacent vertebrae, especially in the elderly with heavy work.^[27]^ TLK was affected by SS, which was determined by PI and PT. The pelvis is critical factor for sagittal alignment and is responsible for retroversion. Based on the relation SS = PI-PT, the amount of pelvic rotation can even approximate PI with sacral endplate horizontal, so individuals with high PI have wider range of adaptation. This movement is very significant, corresponding to an increase of PT correlated to back pain and disability.^[28,29]^ Therefore, the retroversion of pelvic, in association with proximal spine forward leaning and even dorsal hin and knee flexion, analogue to sitting and resulting to capacity over-consumption. However, no relevance between TLK and clinical outcomes suggests that TLK puts little impact on quality of life in short-term and adequate decompression on LSS is fundamental method.

Age-related osteoporosis, with sparse trabecular and fragile cortex, can induce thoracolumbar spine deformities, resulting in kyphosis, shortened body length, and trunk anteversion, especially based on dorsally mismatched elastic modulus caused by instrument.^[30]^ Yagi et al^[31]^ elucidated the role of bone strength for developing proximal junctional kyphosis in 113 ASD with 2-year follow-up, they found osteoporosis was a significant risk factor for kyphosis (OR = 6.4) and suggested surgeons should consider prophylactic treatments when correcting ASD with low bone mineral density. In this study, the patients with severe osteoporosis were as almost 10 times as patients without osteoporosis in suffering from DTLK after instrument-free on TLK, which is inconsistent with Yagi et al. Karikari and Metz^[32]^ reported osteoporotic patients were at risk of developing vertebral fractures and instrumentation failure, indicating it is imperative to optimize bone health with medical therapies such as vitamin D, calcium, bisphosphonates, and parathyroid hormone.

The study firstly identifies the efficacy on DTLK decrease by only performing PLIF on LSS, which can propose a new mentality on treatment DTLK and provide evidence for surgeon for pre-operative conversation. The result puts a further explanation on the interaction between the focal deformity and whole spino-pelvic alignment. It also emphasizes the superiority of shorter-level fixation, supplementing and riching the theory of precision treatment. There were some limitations to be mentioned. Firstly, the sample in both groups is so small and a prospective, larger cohort with longer follow up will strengthen the conclusion. It cannot provide surgical strategy for DTLK with LSS or stability in upper lumbar level (T12-L1 or L1-L2) where the instrument and fusion is inevitable. In addition, the results are not suitable for coronal deformity such as degenerative lumbar scoliosis or rigid TLK such as fracture-derived kyphosis, ankylosing spondylitis, or Scheuermann disease.

## 5 Conclusion

DTLK patients get improved by a mean of 8.9 ± 11.5 (°) even if PLIF is only performed for LSS through a short-term follow up. TK and TLK both decreased at final compared to baseline. TLK and clinical parameters are comparably affected at last whether L5 or S1 was selected as LIV except a larger PT in S1 group. The clinical outcomes are indifferent in patients with corrected and uncorrected TLK. PI and pelvic version can affect postoperative TLK and the cases with severe osteoporosis will impede TLK correction. The finding of this study, to a certain extent, supplement the compensatory mechanism of spino-pelvic alignment.

## Acknowledgments

We acknowledge Houshan Lv who contributed towards the study by making substantial contributions to the design and the acquisition of data.

## Author contributions

**Conceptualization:** Shuai Xu, Haiying Liu.

**Data curation:** Shuai Xu, Zhenqi Zhu.

**Formal analysis:** Shuai Xu, Hongguang Zhang.

**Investigation:** Yan Liang.

**Methodology:** Shuai Xu, Chen Guo, Hongguang Zhang.

**Project administration:** Yan Liang, Haiying Liu.

**Resources:** Shuai Xu, Yan Liang.

**Software:** Shuai Xu, Chen Guo.

**Validation:** Chen Guo.

**Visualization:** Haiying Liu.

**Writing – original draft:** Shuai Xu, Hongguang Zhang.

**Writing – review & editing:** Chen Guo, Hongguang Zhang, Haiying Liu.
